# Supplementary material for: Do you take off your mask correctly? A survey during COVID-19 pandemic in Ningbo, China
Source: PLoS One. 2022 Dec 15;17(12):e0279093. doi: 10.1371/journal.pone.0279093 (PMC9754245; doi:10.1371/journal.pone.0279093)
Supplement: Attachment — Submitted filename: Response to Reviewers.docx [file pone.0279093.s001.docx]

Dear reviewers:

Thank you for your kind comments regarding our manuscript (Do you take off your mask correctly? a survey during COVID-19 pandemic in Ningbo, China).

These valuable comments improved the manuscript. We answered your questions and provided detailed accounts of the changes below. The primary corrections are marked in blue in the revised version.

Journal Requirements:

1. Please ensure that your manuscript meets PLOS ONE's style requirements, including those for file naming.

Thank you for your suggestion. We made sure the manuscript met the stylistic requirements of PLOS ONE.

2. Please review your reference list to ensure that it is complete and correct. If you have cited papers that have been retracted, please include the rationale for doing so in the manuscript text, or remove these references and replace them with relevant current references. Any changes to the reference list should be mentioned in the rebuttal letter that accompanies your revised manuscript. If you need to cite a retracted article, indicate the article’s retracted status in the References list and also include a citation and full reference for the retraction notice.

Thank you for your suggestion. We checked the reference list to ensure it was complete and correct. No retracted papers were cited in the references.

Reviewer #1:

Abstract: Needs to be curtailed and specific. Include the type of study undertaken for this research.

Thank you for your suggestion. We reworked the abstract as requested by PLOS ONE's requirements to be concise and specific, and we added the type of study. The abstract is as follows:

Guidelines and recommendations from public health authorities related to face masks have been essential for containing the COVID-19 pandemic. A cross-sectional survey was conducted in Ningbo City, China, from April 8 to 12, 2022. We assessed the behavioral differences and correlates of mask usage, primarily mask-removal. We examined public mask-wearing behavior during on-site COVID-19 nucleic acid detection. The survey instrument was developed based on the guidelines issued by the World Health Organization and consisted of demographics, mask-wearing knowledge, and behavior. We analyzed data from 1180 participants; 73.2% demonstrated good mask-wearing knowledge. However, regarding mask-wearing behavior, only 53.7% knew the correct way to remove a mask; 70.3% maintained hand hygiene after touching the outside. Binary logistic regression analyses revealed that health prevention knowledge and free mask distribution were positively associated with two types of mask-wearing behaviors. Most participants used masks during the COVID-19 pandemic; however, mask-removal and hand hygiene were neglected when touching the outside of the mask. More attention must be paid to mask-removal and hand hygiene details. Local health authorities should consider introducing the free distribution of masks.

Introduction and discussion can be strengthened by including the following points:

1. include a sentence on origin, transmission of COVID-19 (refer and cite: doi: 10.1136/postgradmedj-2020-138234)

2. The role of government in combatting COVID-19 in comparison with the other regions (refer and cite: doi: 10.3389/fpubh.2022.844333).

3. The role of social environmental predictors of COVID-19 in general (refer and cite: doi: 10.3390/vaccines10101749)

4. The effect of vaccine hesitancy in COVID-19 and its effects in trust in science (refer and cite: doi: 10.3390/vaccines9101064)

5. The calibre of predictors in COVID-19 (refer and cite: doi: 10.1136/postgradmedj-2021-141365)

Thank you for the references; these are helpful to our manuscript. We quoted the literature in the introduction and discussion.

First, we supplemented the introduction (doi: 10.1136/postgradmedj-2020-138234), emphasizing the social impact of the COVID-19 epidemic. Then, we discussed the positive effects of social media on health literacy behavior (doi: 10.3389/fpubh.2022.844333; doi: 10.3390/vaccines10101749; doi: 10.3390/vaccines9101064). Internet users had the highest trust in public opinion issued by national and local governments. The government should take advantage of this by actively publicizing positive public opinion and performing appropriate health education.

Materials and methods: Explain more in detail about the survey, the target population, inclusion and exclusion criteria, if any missed cases were included?

Thank you for your suggestion. We explained the survey in detail, including selection criteria; there were no missing cases. This cross-sectional survey was conducted from April 8 to 12, 2022. Due to SARS-CoV-2 infections in Ningbo, the corresponding area carried out nucleic acid testing for all staff. We investigated on-site during this period. Inclusion criteria were as follows: age over 16 years who agreed to participate and possessed the ability to read and write in Chinese. There were no exclusion criteria. Participants scanned a QR code of the questionnaire while waiting for nucleic acid sampling. The data collection was performed online. We recruited 1,180 participants. Finally, 1,180 people were analyzed with no missed cases.

Statistics: divide the covariates into dependent and independent variables.

Mention if bias was generated, and if yes, how was it limited/regressed.

The importance of choosing the statistical relevance should be addressed.

Thank you for your suggestion. We describe the statistical methods, dependent and independent variables, and how we limit bias. The dependent variables were health prevention behavior of mask-wearing measured using the following questions: During the nucleic acid sampling process, how do you take off your mask; do you perform hand hygiene if you touch the outside of the mask? The independent variables included demographic variables (i.e., gender, age, education, residence, professional, free mask distribution, control area) and health prevention knowledge. The data was analyzed using SPSS™ for Windows, version 23.0, and the parallel mediation effects analysis was conducted using the Hayes approach (2013) and PROCESS Macro by performing a bias-corrected bootstrap procedure. One-way analysis of variance was performed with demographic characteristics and health prevention knowledge as independent variables and health prevention behaviors as dependent variables. Finally, binary logistic regression analyses were used to evaluate individual characteristics, and health prevention knowledge levels were related to health prevention behaviors. Odds ratios and 95% confidence intervals were used to quantify the associations between variables and behaviors. The statistical significance level was set at p < 0.05 (two-sided).

Limitations and strengths need to be included in the end of discussion.

At the end of the discussion, we added strengths and limitations. This study took advantage of the large-scale detection of RNA to perform an extensive survey of mask knowledge and behavior, to reveal defects in mask-removal behavior, and to analyze the predictive factors. The results indicate the validity of masks during the acute phase of the pandemic. There are some limitations. First, the study participants were drawn from only one city, and the findings might not be generalizable to other locations. The study did not explore the relationship between adverse behaviors and the incidence of positive cases of COVID-19. Finally, the behaviors were self-reported, introducing reporting bias. Overall, generalization of the results should be regarded with caution.

Reviewer #2: The article needs minor revision in the form of grammarly check and elaborate the statistical methods more in detail in the section.The introduction, discussion and conclusion should highlight the importance of COVID-19 literature instead of directly focusing on the results of your study.

Thank you for allowing me to revise the manuscript. We engaged a professional company to polish the English and fix the grammatical errors. The certificate of language editing as follows.

We elaborated on the statistical methods, listed the dependent and independent variables, and used binary regression analysis to obtain the results. We also described how to reduce bias.

In the introduction, we highlighted the impact of COVID-19 and discussed how countries respond to the outbreak. In the discussion section, we emphasized the significance of the research and the implications for the government. The government should enhance the power and influence of information dissemination, news reporting, and information dissemination. The government should also perform social education and implementation of COVID-19 prevention and safety measures. Finally, we analyzed the advantages and disadvantages of this research.

Yours faithfully,

Nurse Ma
